# Exploring the principles of logotherapy in genetic counseling: Enhancing decision‐making, adaptation, and justice

**DOI:** 10.1002/jgc4.70165

**Published:** 2026-01-06

**Authors:** Nour Chanouha, Anna Chassevent, Ellen F. Macnamara, Kendra Schaa, Renata Thoeny, Janeta Tansey

**Affiliations:** ^1^ Department of Obstetrics & Gynecology University of Iowa Health Care Iowa City Iowa USA; ^2^ Department of Developmental Neurology Kennedy Krieger Institute Baltimore Maryland USA; ^3^ Undiagnosed Diseases Program, National Human Genome Research Institute National Institutes of Health Bethesda Maryland USA; ^4^ Virtue Medicine PC Viktor Frankl Institute of Logotherapy Iowa City Iowa USA

**Keywords:** adaptation, counseling, decision‐making, freedom, genetic counseling, guilt, logotherapy, meaning, suffering, values

## Abstract

Logotherapy is a psychological approach originated by Viktor Frankl, shaped by the thesis that meaning can be discovered even in the most tragic of human circumstances, and through a human's will‐to‐meaning, that individuals have both freedom and responsibility to discover meanings in the moment and ultimate meanings from their unique standpoints and life stories. The authors submit that as the genetic counseling profession straddles the givens of science and disease alongside the openness of human choices, logotherapy is a particularly effective and potent framework for its practitioners. Logotherapy holds space for the painful experiences of guilt, death, and suffering alongside genetic conditions and the responsibility to make decisions about what to do and how to live. Attention to the emerging psychological concerns and possibilities for meaningfulness in these spaces of tension can enhance the core goals of genetic counseling, including informed decision‐making and healthy adaptation. This paper aims to demonstrate the efficacy of meaning‐centered counseling skills to actively empower a patient's narrative and capacities as essential and alongside other health care needs and concerns. This paper focuses on the general approach of logotherapy and highlights two specific techniques: Socratic Dialogue and Dereflection. From the authors' own multiplicity of practice and teaching perspectives in the genetic counseling field, the paper argues that logotherapy is highly applicable to the profession, highly compatible with short‐term counseling interventions and across varied specialties, offering counselors and their diverse patient populations a more meaning‐rich experience of care, respect, and decisional empowerment.

**What is known about this topic**

Proficiency in counseling skills is both a professional and ethical responsibility of genetic counselors and upholds the profession's core values of care and respect for the patient's autonomy, individuality, welfare, and freedom. Logotherapy is a meaning‐centered therapeutic approach that has been widely applied across healthcare settings and diverse cultures to improve mental and physical well‐being (Batthyány, 2016, pp. 53–74).

**What this paper adds to the topic**

The principles of logotherapy, grounded in the human search for meaning, align with the circumstances and needs of many patients who present for genetic counseling. Logotherapeutic techniques focus on supporting patient strengths and meaning in adversity, while also depathologizing the reality of human uncertainty and suffering. Logotherapy can be integrated with other counseling approaches and builds upon core counseling skills of genetic counselors. We outline the application of logotherapy as a meaning‐centered approach, along with two specific techniques for the practice of genetic counseling.

## 1 INTRODUCTION

In “Psychosocial Genetic Counseling,” Dr. Jon Weil states the following: “The genetic counselor must keep in mind the central importance of the search for meaning and be alert to its many possible manifestations (Weil, 2000, p. 8).” This search for meaning often leads to existential questions about meaning and meaninglessness in life, the possibilities and limits of freedom, and the nature of suffering, dying, and death. In encountering these deep human questions and their normal discomfort, there are associated questions of who or what is responsible for the pain, and in seeking a root cause or agent of responsibility, fear, anger, guilt, and shame may arise. Such feelings are common experiences for the practicing genetic counselor as they facilitate understanding, decision‐making, and support for the patient and family.

Meaning‐making has long been recognized as a central mechanism of psychological adaptation to threat. In her cognitive adaptation theory, Taylor (1983) argues that individuals facing disruptive or stressful events, such as cancer, strive to construct meaning, regain a sense of mastery, and restore self‐esteem as essential adaptive tasks. Recent empirical work supports this theory, demonstrating that meaning‐making predicts better adjustment in chronic illness contexts (e.g., Ferreira‐Valente et al., 2021), and that logotherapy‐based interventions can improve disease acceptance and self‐awareness in patients with serious medical conditions (Mehrizi et al., 2022). These studies contribute to situating logotherapy within a broader social‐behavioral adaptation framework in which meaning‐making plays a central regulatory role.

Logotherapy, with *logos* defined as meaning, is the psychological theory and therapy developed by Viktor Frankl (Bushkin et al., 2021). Logotherapy “puts into a holistic system much of the wisdom of the ages” (Fabry, 2013, p. 14), and is both shaped by and shapes other philosophical and therapeutic approaches that focus on existential concerns, the meaning of life, and coping with suffering. Logotherapy asserts that to be human is to have both freedom and intrinsic meaningfulness. It is a whole‐person philosophy, in which the body, mind, and spirit work in concert toward a will‐to‐meaning. When there are circumstances beyond our control, these are growth opportunities in which meaning is still accessible. Without masochism or nihilism, experiences of suffering, guilt, and death invite attention to where freedom and meaning remain accessible. Logotherapeutic work focuses both on increasing capacity—to be response‐able—and supporting a client's meaningful discovery, choices, and attitude—to be responsible. These themes closely correspond to the needs encountered in genetic counseling, making logotherapy a compelling theory for practice.

Several thought‐leaders in the genetic counseling field such as Seymour Kessler have advocated for the application of a counseling model in genetic counseling practice to deal with the personal meaning of information being shared and provided in different sessions (Kessler, 1997). Additionally, counseling‐oriented practice models such as the Reciprocal‐Engagement Model (REM) have been well described and accepted by many in the genetic counseling profession (Hartmann et al., 2015; Veach et al., 2007). Despite a philosophical acceptance that “being psychosocial” is core to the genetic counseling scope of practice, many studies have demonstrated a lack of its application (Shugar, 2017). Several meta‐analyses found that the dialogue in genetic counseling interactions is more heavily focused on the scientific and biomedical communication of information rather than psychosocial aspects (Meiser et al., 2008; Paul et al., 2015). Hartmann et al. (2015) also highlighted that some genetic counselors feel that psychosocially focused goals can be achieved only on a superficial level in a genetic counseling session and that delving “too far” in that area may be even outside of their scope of practice.

We present logotherapy as a counseling theory that not only addresses the needs of patients navigating existential questions and complex emotions but also equips genetic counselors with skills and techniques to practice at the top of their scope. The application of logotherapy has also been shown to be effective in many other healthcare spaces that overlap with the patient populations served by genetic counselors (Thir & Batthyány, 2016). Those include but are not limited to: palliative care (Eskigülek & Kav, 2024), cancer (Aiello‐Puchol & García‐Alandete, 2025; Barroso et al., 2023; de Meiros et al., 2024; Rezaei et al., 2025), infertility (Mosalanejad & Khodabakshi Koolee, 2013), and cognitive and neurodevelopmental disabilities (Faramarzi & Bavali, 2017; Zekri et al., 2024). The authors of this work have turned to logotherapy to equip us with a wider breadth of techniques to understand and facilitate both challenging patient care and the support/teaching of peers and trainees from a meaning‐rich perspective. Logotherapy has allowed us to invite patients to reflect on the depth of their life stories, their relationships, and the uniqueness of their standpoints. They are invited to courageously engage in difficult conversations, reminded that what they are experiencing is deeply human and greatly matters to them and to us.

Many readers may be familiar with the logotherapeutic themes and techniques presented here within the broader context of meaning‐centered therapy approaches. Logotherapy is in strong alignment with genetic counseling practice‐based competencies, including use of applicable counseling skills and theories to support psychosocial adaptation and patient‐centered decision‐making (Accreditation Council for Genetic Counseling, 2023). Some of the profession's thought‐leaders, Abrams and Kessler (2002), frame genetic counseling as a logotherapeutic process, describing it as a “relationship between real thinking and feeling people,” which “joins us to a common humanity” (Abrams & Kessler, 2002). Further, aspects of logotherapy are congruent with other counseling theories and techniques familiar to genetic counselors, such as solution‐focused, narrative‐focused, and positive psychology, and with ethical topics such as disability care and cultural competency.

Beyond clinical competencies, logotherapy may help genetic counselors fulfill the “professional identity” competency by promoting engagement in their own self‐reflective practice of meaning‐discovery as professionals and in professionalism, for ongoing service, growth and skills‐development for the inclusive, just, equitable, and safe environment for individuals and communities (Accreditation Council for Genetic Counseling, 2023).

## 2 CLINICAL THEORY OF LOGOTHERAPY

### 2.1 Meaning

At the very core of logotherapy is the concept that a person's *Will‐to‐Meaning* is their primary motivation (Frankl, 2006, p. 99). The *Will‐to‐Meaning* refers to the fundamental human drive to find purpose and meaning in life. This meaning‐discovery keeps us resilient and motivated; it is the root of joy and satisfaction. It can be found in one's daily tasks such as service to others, in transcendent experiences such as love and beauty, and in the attitudes one chooses, even in adversity. For Frankl, people do not “make meaning,” meaning is intrinsic to being human. Logotherapeutic conversations help uncover meaning through the exploration of our “conscience” (Fabry, 2013, pp. 57–70). Meaning is specific to both each individual and the time and circumstances of their life in the moment. No two individuals uncover the same meaning, and a single individual may uncover a different meaning at different points in their life. Each person's unique circumstances, relationships, and stories underscore that meaning‐discovery belongs fully to the person's personal conscience work. This concept is seen in genetic counseling patients, where the same genetic information or test result can have a vastly different impact on different patients or the same patient at different times, as life's depth reveals the evolution of what matters. For example, learning of an NF1 variant in his child may be devastating for a father with no family history, but may bring clarity for a father who himself has always wondered why he has so many skin lesions. But even then, genetic counselors must not oversimplify and assume meaning for any patient. The therapeutic aspect of logotherapy is found within the process of uncovering meaning with the patient.

### 2.2 Values

While meanings are unique to an individual, in logotherapy, *values* are commonalities of meanings within a group of people or society (Fabry, 2013, pp. 48–56). For example, medical providers as a group identify principles of autonomy and justice as values (Frankl, 2014, p. 37). As people can belong to many groups (i.e., families, religious groups, social groups, professional groups, ethnic groups), they may prioritize values differently depending on the group they are currently aligned with or the group(s) within which they are living and making choices.

Sometimes values support the discovery of meanings, but personal meanings might also compete or misalign with shared societal or familial values. For example, a patient may say that neurodiversity is important to their understanding of a just society, but also that they personally do not believe they should be asked to parent a child with autism. They *value* neurodiversity, but do not find *personal meaning* in parenting a neurodivergent child at this time. These clashes of values and meanings are one of the reasons that patients may find themselves in tension in a difficult place or decision. Logotherapy holds space for such conflicts, and logotherapists resist seeing such tensions as a problem to be fixed, instead as an opportunity to grow and practice autonomy. This is an opportunity for genetic counselors to foster greater freedom and responsibility, concepts critical to logotherapeutic work, in a patient's will‐to‐meaning.

### 2.3 Growth‐oriented tension

Experiencing tension in difficult situations or decisions in life is an existential reality of being human, not a pathology. When strained in finding understanding, making choices, or resolving a conflict, these are “healthy tensions…stretching from what we are to what we have a vision of becoming (Fabry, 2013, p. 75–76).” For example, consider a parent's drive to search the internet for answers, a diagnosis, or a path forward for their child, unwilling to leave any stone unturned in the search for a responsible next step. Or, perhaps, a patient who is facing a conflict in values and needing to set priorities. For a stuck parent, it could be reminding them that advocating, scheduling appointments, and aiding their child in receiving therapies is meaningful in its brave responsibility, despite uncertainties. One need not understand all possibilities or find a “perfect” solution in order to lean into freedom and responsibility. Logotherapy tolerates tension in the therapeutic session, leaning into the tension within a patient's story, seeing it as a growth edge guiding the patient toward a deeper sense of purpose.

### 2.4 Blows of fate

Patients who present for genetic counseling frequently live with medical conditions, diagnoses, or genetic test results that feel unfair, painful, and meaningless. Patients ask, “why me?” questioning the cards they have been dealt, trying to make sense of life‐changing information. In logotherapy, these situations are referred to as the unavoidability of suffering, or as Blows of Fate: “We must never forget that we may also find meaning in life even when confronted with a hopeless situation, when facing a fate that cannot be changed (Frankl, 2006, p. 112).” These are outside a person's control and may be initially perceived as meaningless, cruel, or absurd. Examples might include a cancer diagnosis or a positive *BRCA1* testing; these are not changeable. These forms of “fate” cannot be healed by medicine or by providing more information, but caring for them as a therapeutic concern supports that life is meaningful even in unavoidable suffering. “When we are no longer able to change a situation—just think of an inoperable cancer—we are challenged to change ourselves (Frankl, 2006, p. 112).”

From a logotherapeutic perspective, blows of fate invite changing one's perspective or attitude toward an openness toward meaning. Logotherapy never asks that people find “the silver lining” in devastating circumstances or claims that there is meaning in the death of a loved one or in the permanent loss of health. Rather, it offers hope for meaning in the face of meaninglessness or suffering, encouraging one to pivot from “why me?” to “what now?” Some patients find a path toward meaning by deciding to act decisively for something that matters to them. Others by practicing mindful and compassionate acceptance, with deep respect for their ongoing capacities to love and choose. Logotherapy supports patients to understand that meaning is never fully lost, either in the present or in their future, even in suffering. When patients and families struggle and find themselves stuck, logotherapy offers a task. They are to search for meaning, listening to their conscience and values, their passions and loves, their relationships of care and perhaps their spiritual guides and beliefs. There are always meaningful choices available alongside their unique sufferings.

It can be difficult as a genetic counselor to sit with a family who is suffering, perhaps feeling one's own suffering or even a sense of guilt that they caused the suffering by sharing a terrible diagnosis. A logotherapeutic approach allows the counselor to bear witness to the suffering as therapeutically meaningful, even in its difficulty, while also providing tools for counseling the patient.

### 2.5 Tragic triad: Suffering, death, guilt

Suffering, guilt, and death are givens in human existence—unavoidable aspects of our lives that Frankl calls the *Tragic Triad* (Frankl, 2014, p. 51). Genetic counselors regularly hold this space with patients. Logotherapy posits that the stance one takes toward the tragic triad—that is, one's response‐ability rather than denial or helpless victimization—provides a meaningful way forward.

Suffering may be physical pain or disability, but it can also include existential or emotional suffering. It is not just one's own, but sharing in the suffering of others and feeling it with deep care, and perhaps even anger at the injustice of it all. “*I/They did not deserve this suffering* – *it is wrong and unfair*.” These are normal human beliefs and emotions; life often is deeply unfair. But life remains full of meaning. Famously, Frankl paraphrases Friedrich Nietzsche in saying, “Those that have a ‘why’ to live can bear with almost any ‘how’ (Frankl, 2006).”

Underlying much suffering is the fear of death, where in facing dying, we wonder: *Has my life been meaningful? Did it matter?* For genetic counselors, death is often encountered in the context of providing a lethal or potential life‐limiting diagnosis. This can create anxiety and decisional paralysis around death and dying. As an existential approach, logotherapy reminds us that death is simply a reality of life, realizing and recognizing meaningfulness, even in our finiteness. Practically, this may look like exploring how a patient wants to be remembered, creating a plan that makes those memories visible for sharing. It may also look like choices to bring focus away from the illness or diagnosis, not as denial, but emphasizing that the diagnosis itself will not be allowed to obscure the chosen meanings of dying/death. When the genetic counselor shares information about death, they should recognize that they are laying the foundation for discovering meaning through this suffering.

Guilt is another existential given. It might be valid/true guilt when the person had some degree of control over their actions and did wrongly, or invalid/false when the person had no control but still harbors a belief that they are responsible. Both forms of guilt often show up during genetic counseling sessions. Examples of valid guilt include a patient's remorse when they delayed cancer screening despite a known *MSH2* pathogenic variant, found late with an advanced‐stage colon cancer, or for the parents who had an increased genetic risk, and in choosing to conceive naturally, have a child with a genetic condition. Guilt may come in degrees; that is, one might believe the choice made was right but still feel guilt for certain harms that were not avoided. Examples of false guilt might include the mother who blames herself for her child's condition because she forgot a dose of prenatal vitamin, or the couple who question their use of fertility treatment in causing their child's de novo genetic condition.

It should be noted that to the person experiencing guilt, it is difficult to tease apart whether the guilt is true or false. It takes skilled genetic counseling to support a patient in exploring the facts and feelings behind their guilt. When a patient is experiencing true guilt, genetic counselors must resist an urge, albeit empathetic, to simply absolve the patient's guilt and responsibility. Instead, they can offer nonjudgmental compassion, through both verbal and non‐verbal behaviors, to help promote the patient's self‐acceptance and development of compassion for themselves. Genetic counselors can explore self‐forgiveness as a therapeutic process, a stepping‐stone that acknowledges the growth‐edges that come with guilt and the courage of regret and repair. Guilt can be tapped for personal growth, informing future decisions and behaviors through greater responsibility. Just as with suffering and death, logotherapy supports the patient's work with guilt as a process.

### 2.6 Freedom and responsibility

Foundational to logotherapy is the work of owning freedom and responsibility. Humans are not perfectly free, but where we are and can be free, meaning and values support us to be responsible. Here, responsibility is literally, “the ability to respond” (response‐ability) to meaning offerings in a situation, guided by the values of the community and the person's own conscience. Understanding where we do have meaning‐rich freedom and responsibility offers a path through the limits of freedom created by suffering, guilt, and death. In suffering, people can find meaning by making responsible choices where choices are available, but it is also important to see where there is an absence of responsibility—either because it infringes on another's freedom or because we are not free ourselves in that space.

Freedom and responsibility are vital to living meaningfully; helping patients identify where they still have freedom, what they are truly responsible for, and why/how they respond is the ethical heart of genetic counseling. Freedom and responsibility still exist in uncertainty. Uncertainty, much like the tragic triad, is a normal part of being human; it is how patients respond to and the choices they make toward meaning through uncertainty that provides fertile ground for logotherapeutic genetic counseling. Genetic counselors can offer grace for decisions made with old information; they can hold space for navigating the uncertainties even toward dying and death, and they can explore their patients' meanings and values, including the strengths of perseverance, forgiveness, loving‐kindness, and spirituality.

Genetic counselors might reflect on their own professional service and care by searching for where their own responsibility lies in the therapeutic encounter. We have already noted that it is not the counselor's responsibility to offer shallow reassurances to guilt—this would interfere with the patient's freedom to think and feel through the uncertainty. It could also never be the genetic counselor's responsibility to fully prevent or cure suffering, as surely there is no way to take the pain away from a family who learned their child has a terminal genetic condition. Nor should genetic counselors prescribe specific meanings for their patients. Rather, they bear witness to their unavoidable suffering and guide them to the choices that remain available.

### 2.7 The noetic spirit, self‐transcendence, and ultimate meaning

Frankl's “dimensional ontology” conceptualizes that humans operate in three dimensions: soma (body), psyche (personality/mind), and noesis (spirit). These dimensions are co‐occurring and holistic. The mind and the body are not separate and are often difficult to differentiate from each other in real‐life phenomena. The noetic (or noölogical), where conscience and its will‐to‐meaning work resides, is never broken but may be suppressed, unconscious, or quiet. As a psychological approach, logotherapy acknowledges and finds therapies for the soma and psyche when they are disabled or sick, while tapping the noetic as an inner resource for building the health of the whole person (Fabry, 1988).

“Depression” may be an overlapping combination of soma, psyche, and noetic suffering, depending on the person and the circumstances. In genetic counseling, patients struggle with anger, pain, sorrow, and/or guilt as a result of diagnoses that are not subject to medical fixes. Logotherapy, stimulating will‐to‐meaning, invites the human spirit to both help and transcend the sufferings of the soma‐psyche. Self‐transcendence means reaching beyond oneself to discover meaning, often found in creativity and/or love. For example, starting a support group for other mothers with pregnancy loss after losing their own child is a self‐transcendent practice of meaning. Or consider an adult child who lovingly cares for his father with Huntington's, knowing that his fate might resemble that of his father. In counseling, we listen for deep ways of knowing toward self‐transcendence. Frankl liked to call logotherapy a *height‐psychology*, contrasted with depth‐psychology; that is, human beings reaching upward, beyond themselves and their suffering. “Life transcends itself not in ‘length’…but in ‘height’—by fulfilling values (Frankl, 1963, p. 78).”

A patient's noetic dimension can include not only meaning in the moment but also *Ultimate Meaning* (Frankl, 2000). Logotherapy is unique in many psychological traditions in honoring that religion, spirituality, and cultural traditions can be powerful and therapeutic sources of personal meaning. The genetic counselor can support patients as they use religious or spiritual practices, rites, texts, and community to understand what matters to them. Ultimate Meaning need not be theistic. There are many secular, spiritual possibilities for a person and/or their community's identified ultimate meanings, including but not limited to compassion, loving‐kindness, higher consciousness, mindfulness, and nature.

Logotherapy reinforces an inclusive model of genetic counseling practice that attends to the cultural beliefs, expectations, and norms of the communities being served. The values central to logotherapy, which are experienced uniquely, are broadly shared across cultures and communities, allowing genetic counselors to approach patients with a sense of mutual understanding and curiosity for the unique circumstances a patient faces. As previously outlined, logotherapists do not put meanings or prescribe values to patients but work to uncover the patient's meanings and values based on their culture and experiences. Logotherapy recognizes that no two people ever have the same meaning and respects meaning‐discovery as valid in itself.

## 3 LOGOTHERAPY IN PRACTICE, WITH TWO TECHNIQUES

This section highlights the general approach to logotherapy and two techniques: Dereflection and Socratic Dialogue.^1^ These are two of the primary interventions central to the practice of logotherapy. When a patient is stuck in a difficult situation, with overwhelming emotions, or facing a complex decision, the techniques explored below are helpful in meeting the mutual goals of shared decision‐making and adaptation present in a genetic counseling consult.

### 3.1 Listening and discovering meaning together

Listening is essential to practicing logotherapeutically; we “must listen for the message, not simply to the sounds (Lukas, 2019, p. 5).” When considering how to incorporate logotherapy into genetic counseling practice, an important first step is to listen for “meaning statements” in conversations with patients and draw attention to them, as patients may not be fully aware of their importance in decision‐making. Genetic counselors can hone in on the motivations that patients give to focus on the meanings and values within them. When a patient says, “I'm tired of taking my medication and going to all these screening appointments,” a genetic counselor can pause and consider what value judgment is motivating this statement. Being curious and asking a follow‐up question to explore the values at play and then reflecting it back is a successful strategy for clarifying an individual's motivation and meaning. This practice is also fundamental to successfully utilizing the specific techniques described later.

There are three general categories of values in logotherapy: creative, experiential, and attitudinal values. Creative values are ways that humans find meaning by creating or doing. This could be doing something artistic, organizing something well, or even creating a new build in Roblox. Experiential values are things that bring one value just by experiencing them: the joy that a parent gets from seeing their child take that first step after years of therapy, the experience of going to a community block party together, the beauty of a concert, or the majesty of watching a sunset. Attitudinal values are one's meaningful choice of attitude, often in spite of suffering: for example, an attitude of bravery, kindness, gentleness, or humor.

Genetic counselors listen for meaning in statements like, “We used to be able to go camping and on vacation with our daughter, but now with all her needs, I'm stuck in the house all day.” The patient is expressing a loss of experiential values and freedom. Genetic counselors can reflect that these experiences together were powerful, giving purpose to the family, and now they are a source of suffering. After exploring whether the genetic counselor has accurately understood the family's meanings and values, they can work with the family to make new plans that are still meaningful, even if different. Genetic counselors can guide the family toward choices that retain freedom and hold experiential value while acknowledging the tragedy of suffering. It is possible that listening and discovering meaning with a patient is all that is needed at a given time. It is also possible that through listening, the genetic counselor may identify opportunities to try a further technique, such as Dereflection or Socratic Dialogue.

### 3.2 Dereflection

Worry, fear, and anxiety are common emotions present in many genetic counseling sessions. Often these emotions are a natural reaction to the indication, test result, or diagnosis. However, if these emotions persist at high levels, it can impede one's daily life, well‐being, and decisional capacity (Llera & Newman, 2020). If a patient is ruminating on hypothetical future outcomes, it can create a sense that one is a helpless victim of their circumstances, rather than a person with freedoms in every circumstance. This can, in turn, emphasize feelings of indecision.

The technique of Dereflection encourages an individual to redirect their focus away from fear, toward where there is still freedom to pursue goals shaped by meaning and value. In Dereflection, rumination is identified and corralled, turning instead to thoughts, feelings, and choices that are meaningful and free. Dereflection can help patients regain a sense of autonomy and decisiveness by allowing them to focus on the freedoms that they have in the present and future, rather than the unknowns that they are trying—futilely—to manage.

#### 3.2.1 Case vignette A

A 34‐year‐old female presents to a prenatal clinic to speak with a genetic counselor about her genetic screening and testing options in pregnancy. She has a history of two prior first trimester miscarriages. During the conversation, the counselor notices that the patient is disengaged.

GC You do not seem interested in speaking about any of these options. Is genetic information about your pregnancy helpful?

Patient There is no point in talking about these options because I know my pregnancy will end before I make it out of this trimester. I am the reason I have had so many miscarriages. I know my stress will cause me to lose this baby too.

GC I hear you. You feel stuck in what seems like the inevitable. How have you been taking care of yourself to try to ease your anxiety?

Patient Well, I started taking yoga and meditation classes, but I hate them! Even though my doctor said I could, I don't drink any caffeine, because sometimes it makes my heart race. I read about IVF, pregnancy, and parenting so that I am prepared for any situation. I am doing everything I can, and I am still failing. I am always anxious.

GC In the past, what kinds of activities brought you joy?

Patient I used to love gardening and reading. I loved getting together with friends for walks or to see movies. But, I don't have time for those things anymore. I haven't seen my friends since I got pregnant this time.

GC It sounds like you are really good at welcoming good things, whether a beautiful garden, or reading stories and ideas or listening to friends. Maybe you are trying too hard to stop stress and what you really need is to bring in more joy?

Patient Maybe.

GC Let's make a list of 2–3 little things that would bring joy into your life. We won't focus on stopping stress, but instead, letting yourself focus on some other activities that matter to you. And no need to try things that you already know don't work for you! This is your life, your joy.

Throughout the beginning of the session, the genetic counselor, utilizing meaning‐focused listening, could sense that the patient was not engaging with their options and opened the conversation up to the patient about whether genetic information was meaningful to them. The genetic counselor tries to help the patient recognize and end their hyper‐reflection on the things that she believes caused her miscarriages, dereflecting into sources of joy or playful exploration. The genetic counselor allows the patient to recognize the freedoms she still has in this moment, which can help dereflect from feeling out of control in uncertainty. The counselor is not asking the patient to actively avoid her anxieties, but rather, allowing her to see the opportunity to find hope in what would otherwise be meaningless worry and suffering. The patient may not be able to “avoid” her anxiety, but the genetic counselor can help her recognize the choices the patient maintains alongside her anxiety. It is important to remember that Dereflection is only one of many tools that can be used in situations such as this, and if a patient continues to mention their anxiety, it may be important to more directly address the source of this anxiety and management options.

### 3.3 Socratic dialogue

Socratic Dialogue is based on the teaching style of Socrates. In logotherapy, Socratic Dialogue helps patients bring into mindful awareness their life's meanings, knowledge, values, and goals. It actively avoids closed counselor responses, such as information sharing or mere paraphrasing/mirroring, and asks open‐ended questions; the counselor is not the expert in the room; rather, they are helping to find and bring forth what is already inside the story of the patient. In logotherapy, this has been compared to the role of a midwife (Fabry, 1988, 2013). This technique intends to listen deeply enough to get beyond superficial or enculturated answers that may sound pleasing or right but are not truly authentic to the patient. Socratic Dialogue generally avoids affirmation or validation as primary counseling responses, inviting patients to be self‐validating as they struggle to answer the counselor's ways of asking “what?” “why?” or “why not?” A successful use of this technique highlights that the patient had the answer inside them all along; all they needed was powerful questions to help them see and say what is true for them, with the confidence that comes with self‐knowing.

#### 3.3.1 Case vignette B

A 26‐year‐old female presents to the cancer clinic to discuss her family history of cancer. She is speaking to the genetic counselor about the impact of her mother's recent cancer diagnosis.

Patient My mom has always been my rock and now she has cancer. I feel our roles are switched.

GC How have your roles changed?

Patient Mom always tried to do things to care for me. Like bringing food or watching my son when he was little. But I just want to sit with my mom. For her to know I'm there for her. And to make jokes. So that she stays positive.

GC Is showing positivity your way?

Patient Yeah, but I keep thinking how bad I am at doing this.

GC How is this bad?

Patient Well…I am not doing anything like cooking or running errands or cleaning her house. That is what she always did for me, and what she does for others going through hard times.

GC So it is a different way of caring. Is that bad?

Patient Sometimes it feels that way.

GC [Silent Listening]

Patient I guess I see how we all appreciated the chores she did for us. I mean, no one besides my family really knows how much time I am spending with my mom since her cancer diagnosis.

GC Does that bother you?

Patient Well…I wonder if people think that I am not doing enough to help my mom.

GC Are their opinions important?

Patient Is it superficial to want them to see me caring for her?

GC Maybe not. How would you figure out what kind of care you want to show your mom now?

Patient Actually one of my best friends used to call me almost every day after I had my son and was going through postpartum depression. I didn't always answer, and then she would text me pictures from our fun college days. Her positivity made that period in my life more bearable, but no one else knew she did that for me.

GC [Silent Listening]

Patient I think only me and my mom can decide what kind of caring is best for our new roles. I don't have to please others. I think I'll ask her if what I'm doing is helpful.

Here the genetic counselor is using a technique of “self‐discovery discourse” (Fabry, 2013, pp. 115–133), asking the patient to do the work. Socratic Dialogue is not always comfortable and takes practice. It may seem like there is a struggle between the patient and counselor in trying to find the answers, but when done properly, it will feel like an active partnership that prioritizes all opportunities for meaning in the patient's life.

#### 3.3.2 Case vignette C

A 30‐year‐old male presents to the neurogenetics clinic for follow‐up after a presymptomatic diagnosis of Huntington Disease. He is speaking with the genetic counselor about how he has been coping with this recent diagnosis and seeing his father navigate his own life with Huntington Disease.

Patient This diagnosis has made me very angry with God. I have found that going to Church, something I used to find a lot of peace in, just surfaces my feelings of anger. I love God, and I want to say I trust Him. But, how could He submit me to a life with this disease? Perhaps He punished me with this diagnosis because I was not a good enough Christian.

GC In your belief system, how do you feel that pain and suffering fit into the bigger picture of life's meaning?

Patient I think pain and suffering can teach us humility and trust. It is hard to see it in the moment, but when you come out on the other side, you can see how much you grew.

GC Have there been other times in the past that you were uncertain about God?

Patient After college, I felt stuck finding a job and was unemployed for a long time. I remember having these thoughts. But then I found my dream job, and I realized that I would not have been able to apply for that job if I had been working elsewhere. That felt like God's divine plan!

GC Does that experience help with your current anger?

Patient Maybe He did not punish me with this diagnosis, but instead this is His way of bringing me even further into His love. I feel his support when I pray before bed and when I read my Bible in the morning. Life with this diagnosis will not be easy, but I know that no matter what I do, God loves me and cares about me.

GC In your religious beliefs, how do you think it is possible for both anger and love for God to coexist?

Patient I suppose. I have always been taught that God can handle our anger. It is alright to question Him, as this is just part of what it is to be human.

GC You feel God's love and support. And you feel angry too, and that's a normal feeling because this diagnosis seems unfair. There's a tension here, right? What is the way forward?

The genetic counselor pushed the patient to share more and go deeper into his thoughts through open, inviting statements and, in this case, where meaning is associated with the patient's freedom to find Ultimate Meaning. With the counselor's questions, they were able to work with past experiential meaning toward attitudinal meaning. His grief and anger about his diagnosis, alongside beliefs about a caring God, exist in his life as a meaningful tension or even paradox, without staying stuck.

## 4 CONCLUSION

Genetic counselors are uniquely positioned to engage in an active partnership with individuals and families through what makes us human—suffering, death, and uncertainty surrounding our health and reproductive future. This requires a skill set beyond knowledge of science and technology, risk calculation, and the ordering of genetic tests, to the development of counseling skills that promote a patient's psychosocial adaptation and/or facilitation of decision‐making. “The choices offered by genetic technology, like most important life choices, are filled with moral, ethical, philosophical, and psychological dilemmas which have few easy or satisfactory solutions” (Kessler, 2013, p. 12). Logotherapeutic techniques offer a way to help patients take responsibility for making a choice that is most meaningful to them, at that moment in their life. These techniques work well alongside many other counseling techniques and communication skills, within an established therapeutic alliance.

Logotherapy reminds genetic counselors and patients that living at risk of a genetic condition, or with a diagnosis, does not determine who we are as human beings. In fact, it is our human spirit—the stance we take toward the biological and psychological influences—that offers a source of freedom, responsibility, and meaning. How genetic counselors support patients in their response to the unique circumstances of their lives is what matters. When establishing the therapeutic relationship with a patient, it is important to share that the genetic counseling skillset extends beyond testing and education. Explicitly stating that this appointment may be used to help patients and families identify their values, draw on sources of hope or meaning, or think about ways to live meaningfully in uncertainty or with a genetic risk or diagnosis can provide helpful context.

Logotherapeutic techniques can be applied across practice settings and offer a path toward more meaningful interactions between genetic counselors and the families they serve. Even brief or single encounters offer genetic counselors opportunities to apply a logotherapeutic framework, with or without techniques, understanding that the impact of such interventions may not be immediately apparent. These approaches often plant a seed for ongoing reflection and change in a patient's perspective or behavior beyond the initial appointment. Research supports that single‐session psychosocial interventions, drawn from various theories, demonstrate a positive effect across outcomes and age groups, including for anxiety, depression, and substance use (Bloom, 2001; Yan, 1985).

A short‐term logotherapeutic intervention may include a genetic counselor introducing principles of freedom and responsibility to a patient following a new diagnosis or as part of informed decision‐making, followed by an exploration of how these principles apply to the patient's life. Additionally, a counselor may employ Socratic Dialogue or modification of attitude to reframe unavoidable suffering that often accompanies a diagnosis. One study found that greater therapist Socratic questioning predicted greater reduction in depressive symptoms among adults from session to session (Braun et al., 2015). Further, opportunity exists to provide patients with meaning‐centered questions as “homework” for further reflection and consideration outside the session.

Following a diagnosis, genetic counselors commonly witness a patient's search for meaning—*Why did this happen to me (my child, or my family member)?* Taking a logotherapeutic approach, a genetic counselor avoids a premature response with a scientific explanation, such as “de novo genetic changes occur as a natural part of biology,” or “this condition is likely due to multifactorial inheritance caused by a combination of lifestyle, environment, and genetic factors.” Instead, a logotherapeutic response invites the patient to explore meaning and agency: “That question of ‘why’ really matters. Let's explore possibilities. It will help you decide how to move forward.”

Certain logotherapeutic techniques, including Socratic Dialogue and Dereflection, require sufficient decisional capacity to engage in discussions about thoughts, beliefs, behaviors, and values. Therefore, these techniques may not be appropriate to use following the immediate delivery of unexpected news or during acute crises. However, as processing occurs and the initial shock of a diagnosis subsides, these techniques can be valuable tools in helping patients make sense of this news and explore meaning. Logotherapeutic practice may also be bound in clinical settings that involve patients who have intellectual disability, psychiatric conditions, or neurological disorders, depending on the capacities of the individual. Yet, opportunity exists to apply logotherapeutic concepts to help family members and caregivers who may accompany these individuals to their appointment. Logotherapy techniques are unlikely to be helpful without the counselor's openness to the belief that life matters and it is meaningful even in suffering.

With a celebration of the uniqueness of each individual, logotherapy is an inclusive counseling approach for culturally and ethically competent genetic counseling. Moreover, the themes of logotherapy apply beyond genetic counselors' work with patients to their professional identity and professionalism. Adopting a logotherapeutic perspective prompts genetic counselors to reflect on their ability to understand and sit with suffering, as well as their freedoms and responsibilities to themselves, colleagues, patients, and the broader society. While they could be paralyzed by fear of a changing health care landscape, including artificial intelligence, genetic counselors can dereflect into their freedom to choose how they shape their practice with an abiding responsibility to “respect a patients' beliefs, inclinations, circumstances, feelings, family relationships, sexual orientation, religion, gender identity, and cultural traditions” as fellow human beings (National Society of Genetic Counselors, Inc., 2017). As Frankl reminds us, “When we are no longer able to change a situation, we are challenged to change ourselves” (Frankl, 2006, pp. 112).

Just as genetic counselors celebrate the uniqueness of each patient, so too should genetic counselors celebrate the uniqueness of their professional identity and their individuality as genetic counselors. A logotherapeutic perspective embraces that the value of genetic counseling lies in counselors' unique positionality and practice to create a space at the nexus of science, medical ethics, and patient stories, to allow patients to know that their lives and choices matter, that they are seen and heard with care and respect. By embracing genetic counselors' freedom to practice with intention, as well as their professional responsibilities to honor their own and patients' individual stories as a matter of care and justice, we affirm the ongoing therapeutic possibilities of genetic counseling.

## AUTHOR CONTRIBUTIONS

Nour Chanouha, Anna Chassevent, Ellen F. Macnamara, Kendra Schaa, and Renata Thoeny: Conceptualization, writing – original draft, and writing – review and editing. Janeta Tansey: Conceptualization, writing – review and editing, and supervision. All authors have read and approved this submission.

## CONFLICT OF INTEREST STATEMENT

No conflicts of interest to disclose.

## ETHICS STATEMENT

Human studies and informed consent: This manuscript does not involve human or animal subjects.

## Data Availability

Data sharing not applicable to this article as no datasets were generated or analyzed during the current study.

## Footnotes

^1^
We have not elaborated on the technique of Paradoxical Intention in this paper, as it is usually most appropriate to more severe mental health conditions of obsessionality or delusion. Paradoxical intention uses the human capacity of self‐detachment or reflexivity, which allows us to step outside of our space of suffering and to examine with more distance where it holds humor or absurdity, so that it can be released (Fabry, 2013, pp. 115–133). To utilize paradoxical intention, the patient is encouraged to imagine (or intend) the worst possible outcome of whatever they fear. By doing this kind of cognitive or behavioral exposure work and inviting humor and perspective, the fear can then be extinguished or reframed into a more appropriate and manageable degree. The goal of the technique is to regain a sense of control over one's emotions and thoughts by creating both distancing and resistance to the power of fear or phobia (Frankl, 2006). There may be occasions for some genetic counselors to use this technique.
